# Traumatic Knee Dislocation: A Retrospective Case Series Evaluating Clinical Presentation, Management, and Outcomes at a Tertiary Care Centre

**DOI:** 10.7759/cureus.118008

**Published:** 2026-10-16

**Authors:** Rahul Rathod, M B Lingayat

**Affiliations:** 1 Orthopedics, Government Medical College &amp; Hospital, Chhatrapati Sambhajinagar, Chhatrapati Sambhajinagar, IND

**Keywords:** anterior cruciate ligament reconstruction, external fixation, knee dislocation, orthopedic emergency, popliteal artery injury, road traffic accident, vascular injury

## Abstract

Background:A knee dislocation occurs when there is a total loss of contact between the femoral and tibial articular surfaces, which is uncommon but disproportionately dangerous. Ligamentous, vascular, and neurological structures are frequently injured together, and outcome hinges on how quickly the injury is recognized and treated.

Objective: This study aimed to describe the clinical presentation, mechanism and direction of traumatic knee dislocation, vascular and neurological status, acute management strategies, need for external fixation or delayed ligament reconstruction, limb salvage, and available short-term outcomes in patients treated at a tertiary care centre.

Methods: This retrospective descriptive case series included seven patients with clinically and radiologically confirmed traumatic knee dislocation managed in the Department of Orthopaedics at a tertiary care centre between 1 January 2025 and 31 January 2026. Demographic characteristics, mechanism of injury, dislocation direction, vascular status, neurological findings, imaging, acute management, subsequent ligament reconstruction, and available follow-up outcomes were extracted from clinical records. Findings are presented descriptively as frequencies and percentages.

Results: All seven patients were male (mean age 39 years, range 27-55 years), and all injuries resulted from road traffic accidents. Anterior dislocation was the most common pattern (4/7, 57.1%), followed by lateral (2/7, 28.6%) and posterior (1/7, 14.3%). One patient (14.3%) presented with absent distal pulses and underwent urgent vascular repair with external fixation, achieving limb salvage. External fixation was required in 2/7 cases (28.6%) and delayed anterior cruciate ligament reconstruction in 2/7 cases (28.6%); the remaining patients were managed conservatively. Successful reduction was achieved in all cases, with no amputation or mortality.

Conclusion: Traumatic knee dislocation requires prompt reduction and systematic neurovascular assessment, with early vascular intervention when compromise is suspected or confirmed. This small retrospective case series demonstrates the heterogeneity of management pathways and highlights the importance of coordinated orthopaedic and vascular care. Long-term functional conclusions are limited by incomplete and non-standardized follow-up.

## Introduction

A knee dislocation occurs when the femoral and tibial articular surfaces lose all contact with one another -- a rare injury, but one that carries an outsized risk to the limb [1-3]. Fewer than 1 in 100 orthopedic injuries fall into this category, and even that figure likely understates the true burden, since a considerable share of dislocated knees reduce spontaneously before the patient ever reaches a hospital [2,4].

Four ligaments hold the joint together -- the anterior and posterior cruciates and the medial and lateral collaterals -- and a true dislocation cannot occur without tearing at least two of these restraints. Compounding the danger, the popliteal artery and common peroneal nerve run directly behind the joint line, occupying a position that leaves them exposed to the same forces that displace the tibia [2,5].

Most cases follow a familiar pattern of high-energy trauma -- road traffic collisions, falls from height, dashboard-type impacts -- though a smaller subset occurs at surprisingly low energy in patients who are severely obese [5,6]. Two classification schemes dominate clinical practice: Kennedy's system [7], which describes the injury by the direction the tibia moves, and Schenck's system, which instead catalogues which ligaments have failed [8].

Of the possible complications, vascular injury is the one clinicians fear most: popliteal artery involvement has been documented in as many as four of every 10 cases, with posterior-pattern dislocations carrying the greatest share of that risk [2,9]. Peroneal nerve injury, producing a foot drop, follows a related logic but tracks more closely with anterior dislocations, affecting somewhere between one in 10 and four in 10 patients [9,10]. Because ischaemia lasting beyond 6-8 hours sharply raises the odds of amputation, the initial workup leaves little room for delay -- reduction first, then repeated vascular checks, then a low threshold for CT angiography [11-13].

The purpose of this retrospective case series was to describe the clinical presentation and management pathways of traumatic knee dislocation at a tertiary care centre. The primary objective was to characterize dislocation pattern, neurovascular status, acute treatment, limb salvage, and early clinical outcomes. Secondary descriptive objectives were to document the use of external fixation, delayed ligament reconstruction, conservative treatment, and available follow-up findings.

## Materials and methods

Study design and setting

This was a retrospective descriptive case series conducted in the Department of Orthopaedics at a tertiary care centre. Seven patients with clinically and radiologically confirmed traumatic knee dislocation treated between 1 January 2025 and 31 January 2026 were included. All cases were consecutive. Cases with head, spine, chest, and abdominal injuries were excluded from the study. 

Data collection

Clinical records and available imaging were reviewed for age, sex, mechanism of injury, side, direction of dislocation, distal vascular status, neurological findings, reduction, vascular intervention, use of external fixation, subsequent ligament reconstruction, conservative treatment, follow-up imaging, limb salvage, mortality, amputation, and available functional observations. Missing variables were not imputed.

Initial assessment and neurovascular evaluation

On presentation, each patient underwent trauma assessment followed by focused examination of the affected limb. Distal pulses, capillary refill, limb perfusion, and documented peroneal nerve function were reviewed from the medical record. Neurovascular examination was repeated after reduction where documented. Ankle brachial index (ABI) was not consistently documented in the retrospective records.

Imaging and vascular investigation

Plain radiographs were used to confirm dislocation and post-reduction alignment. Doppler study was undertaken in every case before and after reduction. Emergency CT angiography or vascular-surgical evaluation was undertaken when vascular compromise was suspected on clinical assessment. MRI was used after stabilization in selected patients to evaluate ligamentous injury and guide delayed reconstruction.

Management

Urgent closed reduction was the first-line intervention in all seven patients. External fixation was used in two patients with high-energy or unstable injury patterns, including the patient requiring vascular repair. One patient with absent distal pulses underwent combined orthopaedic and vascular management with reduction, external fixation, and popliteal artery repair. Two patients underwent delayed anterior cruciate ligament (ACL) reconstruction after MRI-confirmed instability. Three patients were treated conservatively following reduction with bracing/protected mobilization and follow-up.

Outcome assessment

Outcomes assessed from the available records included successful reduction, limb salvage, maintenance of alignment on follow-up radiographs, amputation, mortality, documented neurological complications, and available observations of mobility or knee function. Because standardized functional outcome scores were not reported in the source manuscript, no retrospective score was generated.

Statistical analysis

Given the small sample size and descriptive design, categorical variables were summarized using counts and percentages. No inferential statistical testing or comparative effectiveness analysis was performed.

Ethics

The study was conducted in accordance with institutional ethical requirements for retrospective review. The source manuscript states that retrospective data collection was permitted within the institution's ethics framework and that IRB approval was obtained (IRB no. 285/2026).

## Results

This case series comprised seven patients with traumatic knee dislocation. All patients were male, with ages ranging from 27 to 55 years (mean 39 years). Road traffic accidents were the mechanism of injury in all seven cases (100%), consistent with the high-energy trauma typically associated with knee dislocation. A summary of the seven cases of traumatic knee dislocation, including dislocation type, vascular status at presentation, and management pathway, is presented in Table 1.

**Table 1:** Demographic and clinical characteristics of patients with traumatic knee dislocation (n = 7) The table summarizes the age, sex, mechanism of injury, direction of knee dislocation, vascular status at presentation/post-reduction, and management strategy for each patient included in the case series. Management included closed reduction, external fixation, vascular repair when indicated, conservative treatment, and delayed ligament reconstruction based on the individual clinical presentation. RTA, road traffic accident; MRI, magnetic resonance imaging; ACL, anterior cruciate ligament.

Case	Age/Sex	Mechanism	Dislocation Type	Vascular Status	Management
1	34/M	RTA	Lateral	Pulses present	Urgent closed reduction + external fixation
2	55/M	RTA	Posterior	Pulses absent -- vascular injury	Urgent closed reduction + external fixation + vascular repair
3	29/M	RTA	Anterior	Pulses present	Closed reduction (pre- and post-reduction imaging documented)
4	36/M	RTA	Anterior	Stable post-reduction	Closed reduction → MRI → delayed ACL reconstruction
5	47/M	RTA	Lateral	Pulses present	Closed reduction, conservative → MRI → delayed ACL reconstruction
6	45/M	RTA	Anterior	Pulses present	Closed reduction, conservative management
7	27/M	RTA	Anterior	Pulses present	Closed reduction, conservative management

Dislocation pattern

The direction of dislocation, based on the Kennedy classification, was documented in all seven cases. Anterior dislocation was the most common pattern, seen in four cases (57.1%), followed by lateral dislocation in two cases (28.6%) and posterior dislocation in one case (14.3%), consistent with the distribution generally reported in the literature [6,8] (Figure 1).

**Figure 1:**
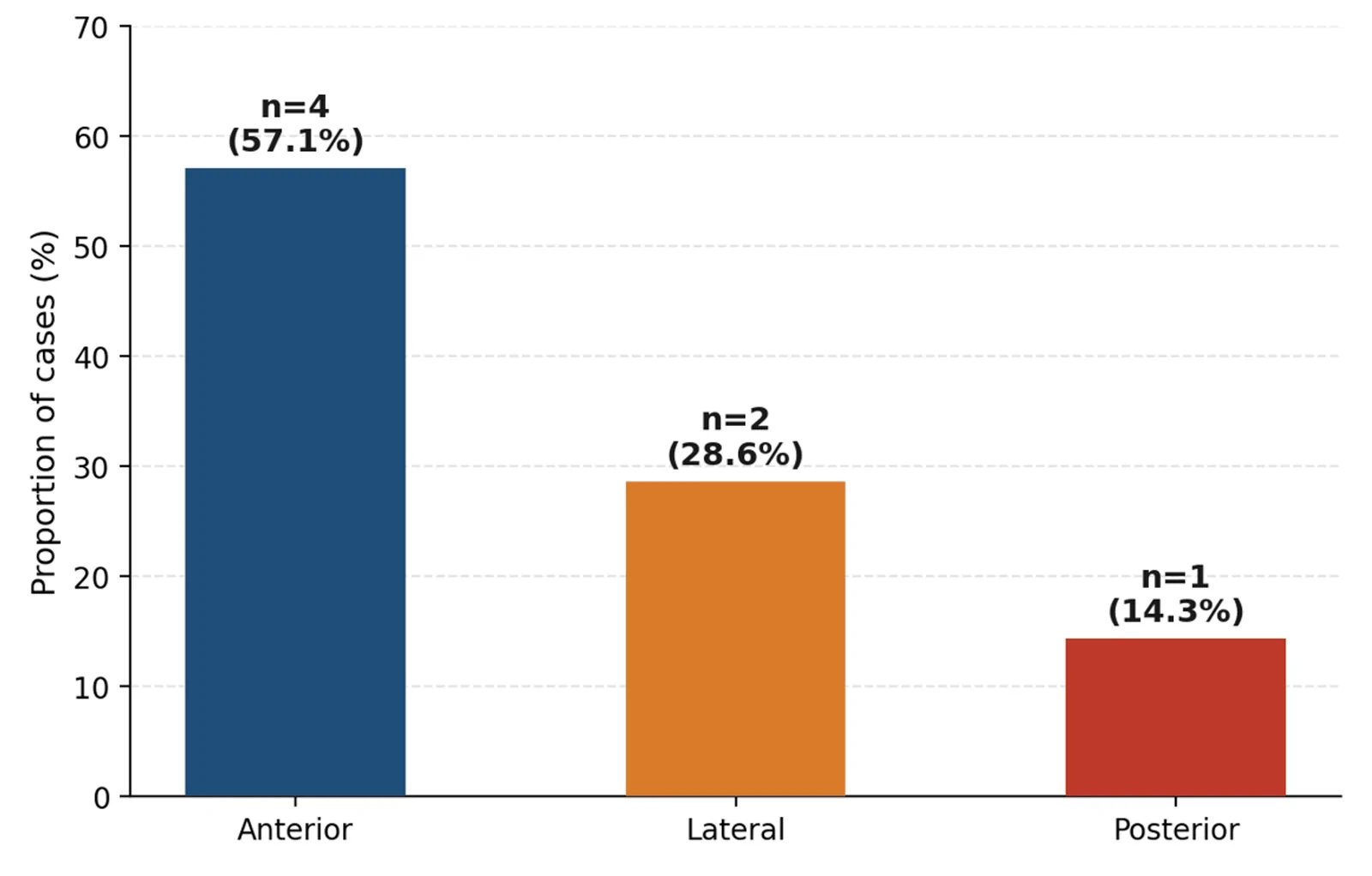
Distribution of dislocation direction Distribution of traumatic knee dislocations according to the direction of dislocation (n = 7). Anterior dislocation was the most common pattern, observed in four patients (57.1%), followed by lateral dislocation in two patients (28.6%) and posterior dislocation in one patient (14.3%).

Vascular assessment and injury

Distal vascular status at presentation was documented in all seven patients. Six patients (85.7%) had palpable distal pulses or a stable vascular examination following reduction, while one patient (14.3%, Case 2, posterior dislocation) presented with absent distal pulses, confirming a limb-threatening popliteal vascular injury. This patient underwent urgent combined orthopaedic reduction, external fixation, and vascular repair (Figures 2-4).

**Figure 2:**
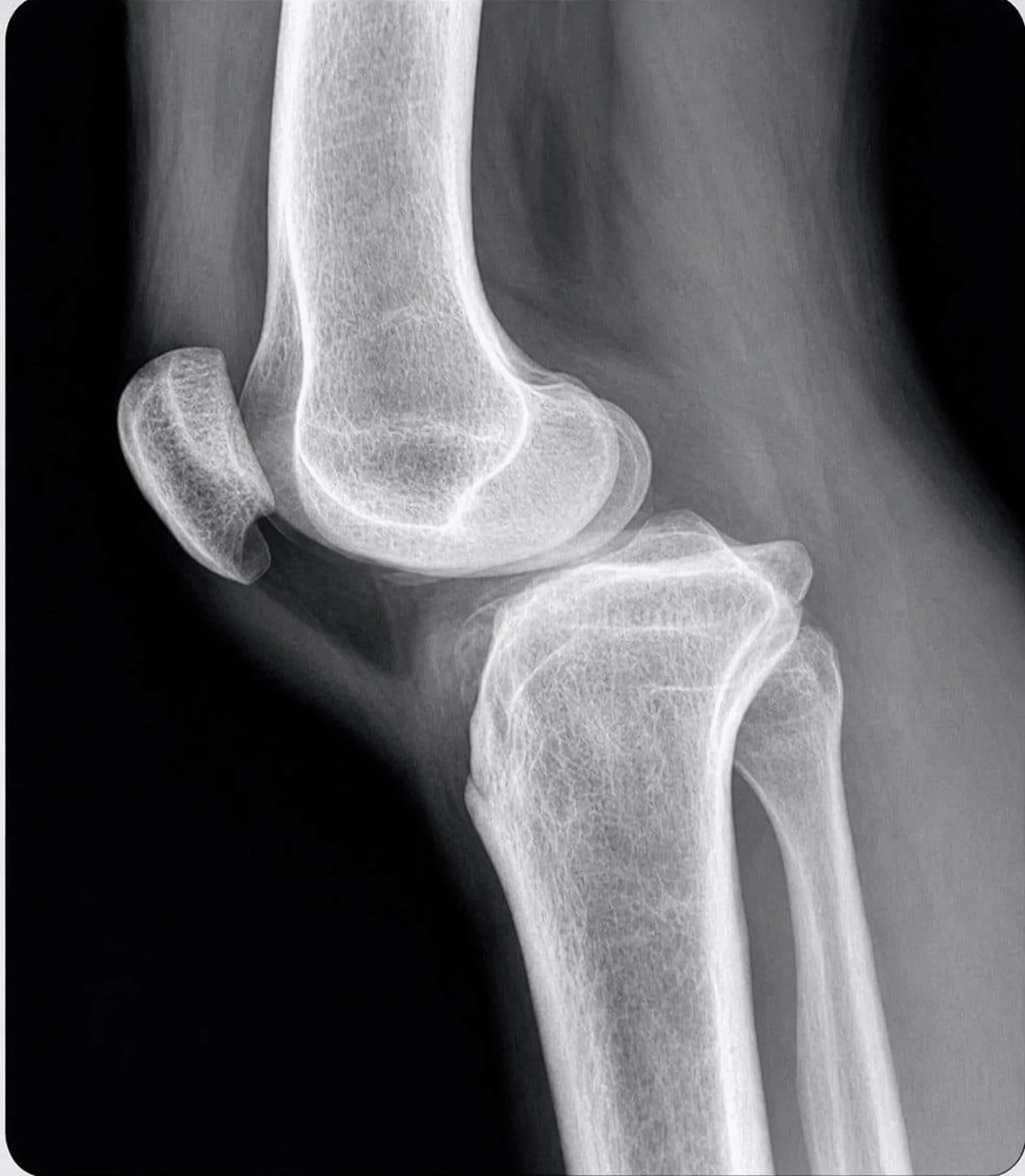
Case 2 (55-year-old male, posterior dislocation): pre-reduction radiographs demonstrating tibiofemoral dislocation in a patient who presented with absent distal pulses Lateral radiograph of the knee demonstrating posterior tibiofemoral dislocation, with posterior displacement of the tibia relative to the distal femur. The patient presented with absent distal pulses, raising concern for an associated vascular injury and necessitating urgent reduction and vascular assessment.

**Figure 3:**
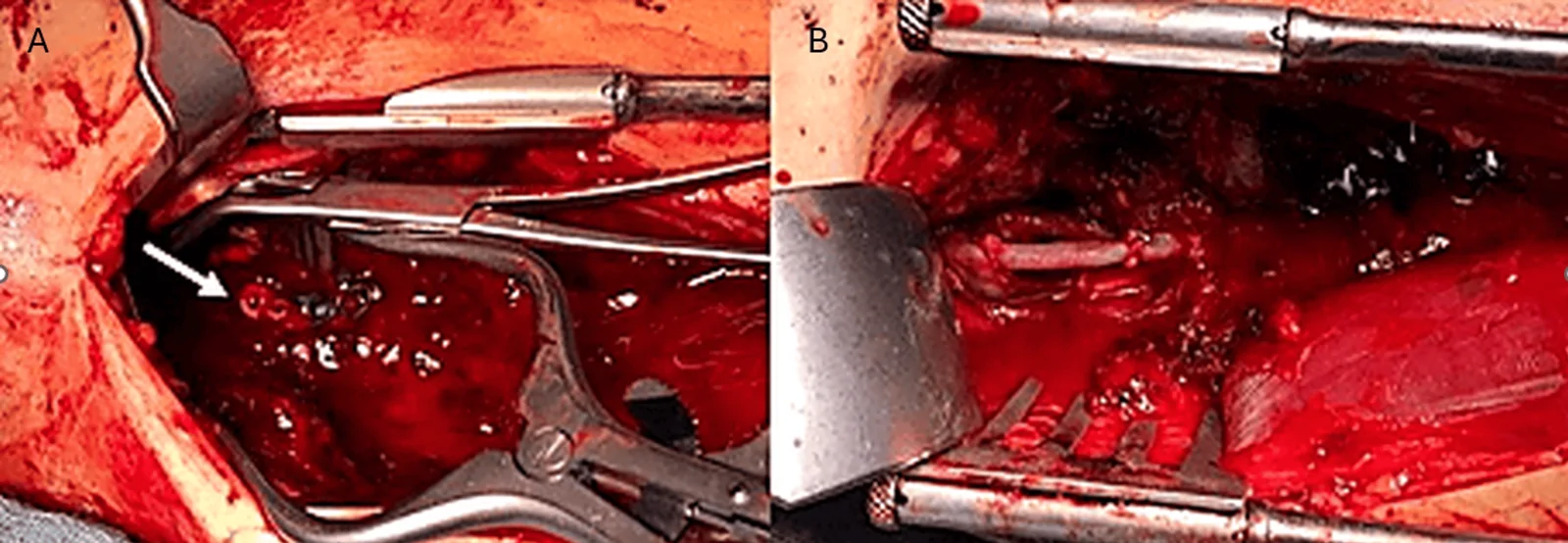
Case 2: Intraoperative photographs demonstrating repair of traumatic popliteal artery injury (A) Intraoperative photograph demonstrating the injured popliteal artery following surgical exposure, with identification of the proximal and distal arterial ends (white arrow). (B) Intraoperative photograph following vascular control with clamps and end-to-end anastomotic repair of the popliteal artery using 7-0 Prolene suture, performed to restore arterial continuity and limb perfusion.

**Figure 4:**
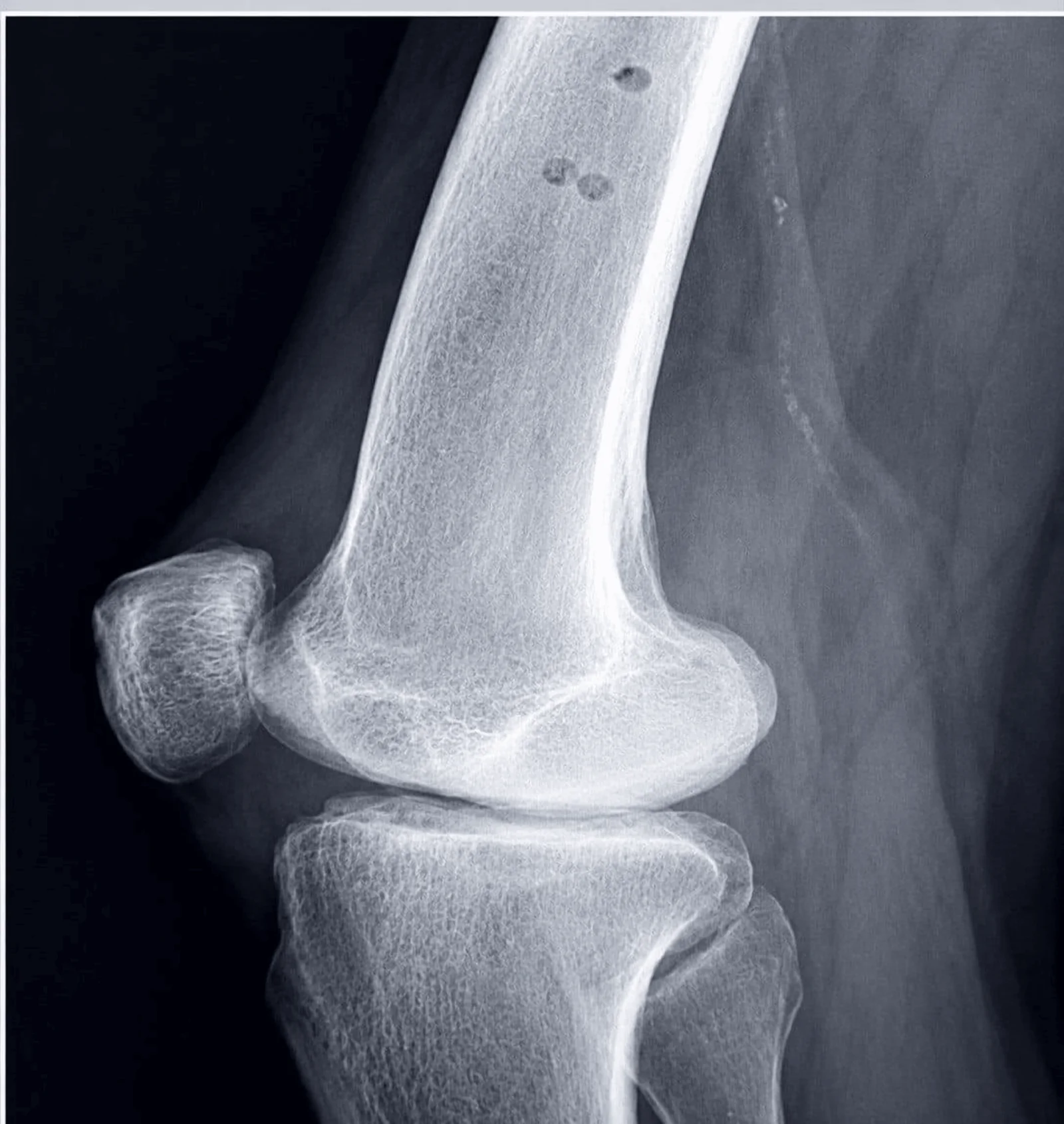
Postoperative lateral radiograph following reduction, external fixation, and vascular repair Postoperative lateral radiograph of the knee in Case 2 following urgent closed reduction, external fixation, and vascular repair, demonstrating restoration and maintenance of tibiofemoral joint alignment with successful limb salvage.

Management

Urgent closed reduction was performed as the first-line emergency intervention in all seven patients (100%). External fixation was required in two cases (28.6%), both involving higher-energy or unstable injury patterns, one combined with vascular repair. Delayed ligamentous reconstruction (ACL repair) following MRI-confirmed instability was performed in two of seven cases (28.6%). The remaining three patients (42.9%) were managed with closed reduction followed by conservative treatment -- bracing, protected range-of-motion exercises, and physiotherapy (Figure 5).

**Figure 5:**
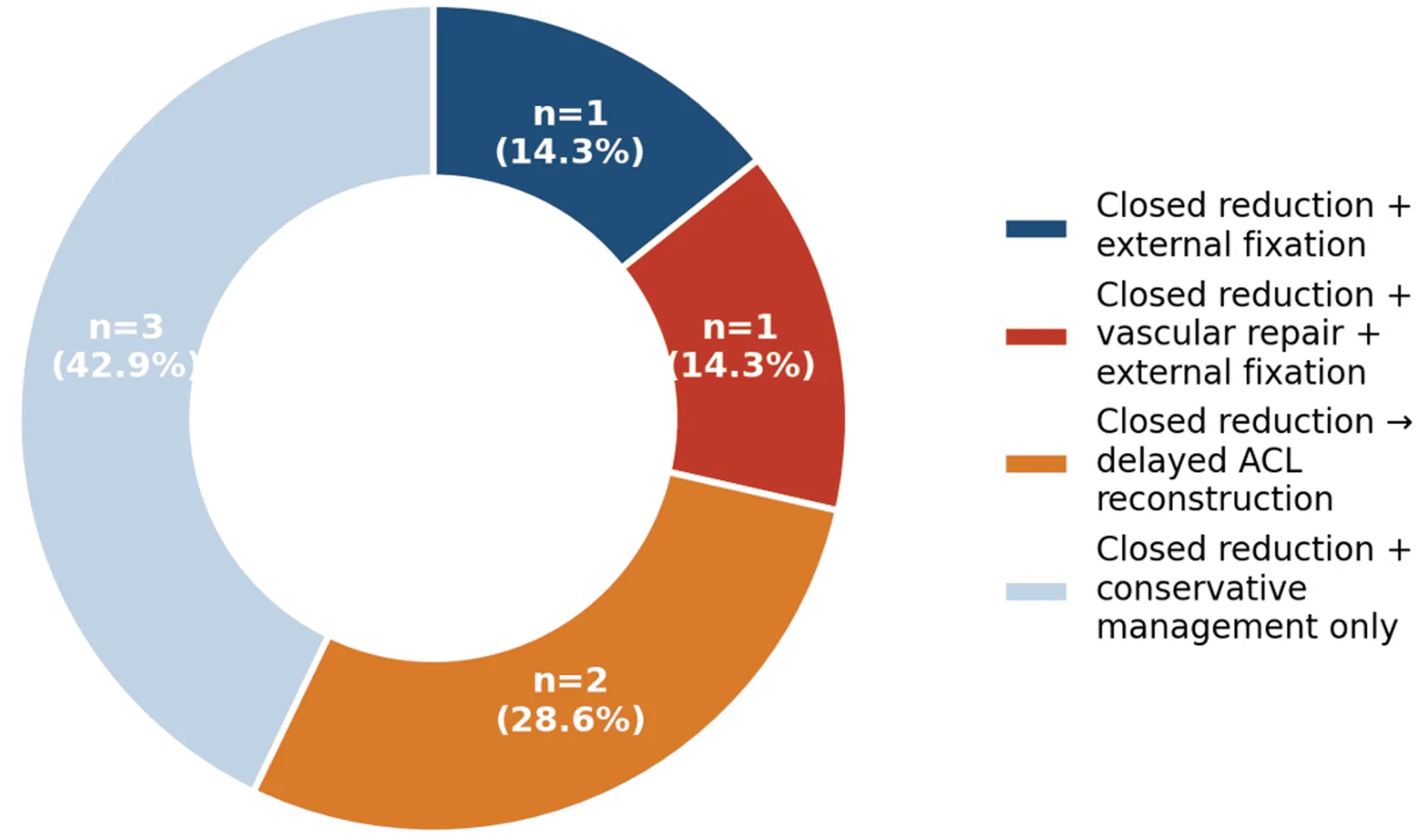
Definitive management pathway adopted for each of the seven cases in this series Distribution of management strategies among patients with traumatic knee dislocation (n = 7). Conservative management following closed reduction was the most commonly employed strategy (42.9%, n = 3), followed by closed reduction with delayed ACL reconstruction (28.6%, n = 2). Closed reduction with external fixation and closed reduction with vascular repair plus external fixation were each performed in 14.3% (n = 1) of patients. ACL, anterior cruciate ligament.

Outcomes

Successful joint reduction was achieved in all seven cases (100%), with no instance of failed closed reduction requiring conversion to open reduction. The patient with vascular compromise (Case 2) achieved limb salvage without amputation following timely vascular repair. Follow-up radiographs documented maintained joint congruity in six of seven cases (85.7%). Functional outcome data (range-of-motion or gait/walking video) were documented for three of seven patients (42.9%), each showing satisfactory restoration of knee mobility and ambulatory function. No amputations, deaths, or documented cases of compartment syndrome or persistent peroneal nerve palsy occurred within this series (Table 2). 

**Table 2:** Summary statistics for the case series (n = 7) Summary of demographic characteristics, mechanism of injury, dislocation pattern, vascular status, management strategies, and clinical outcomes in patients with traumatic knee dislocation (n = 7). ACL, anterior cruciate ligament; n, number of patients; %, percentage.

Parameter	n	%
Total cases	7	100
Male sex	7	100
Mechanism -- Road traffic accident	7	100
Anterior dislocation	4	57.1
Lateral dislocation	2	28.6
Posterior dislocation	1	14.3
Pulses present/stable post-reduction	6	85.7
Vascular injury requiring operative repair	1	14.3
Managed with external fixation	2	28.6
Managed with closed reduction alone (no fixation/repair)	5	71.4
Delayed ligament (ACL) reconstruction performed	2	28.6
Purely conservative management (no operative fixation/repair)	3	42.9
Amputation	0	0
Mortality	0	0

Illustrative case 1

Case 1 is a 34-year-old male patient with post-traumatic lateral dislocation of the right knee managed by urgent closed reduction and external fixation (Figure 6).

**Figure 6:**
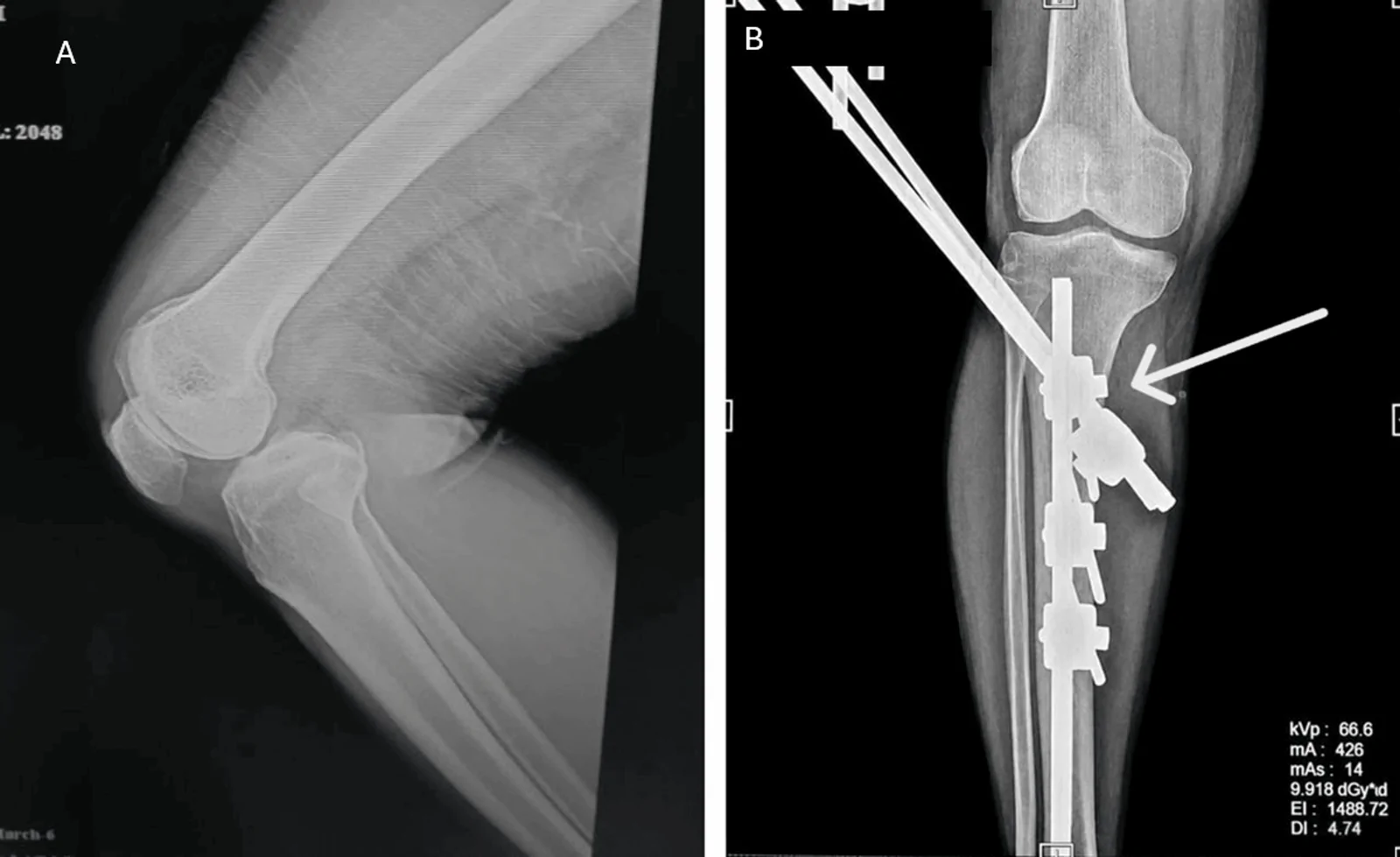
Case 1 (34-year-old male with lateral knee dislocation): Pre-reduction and post-reduction radiographs following urgent closed reduction and external fixation (A) Pre-reduction lateral radiograph of the right knee demonstrating tibiofemoral dislocation. (B) Post-reduction anteroposterior radiograph following urgent closed reduction and external fixation, demonstrating restoration of tibiofemoral alignment with the external fixation construct in situ (white arrow).

## Discussion

Knee dislocation remains an uncommon but limb-threatening injury, and this case series adds to the limited body of Indian tertiary-care experience with this condition. All seven patients in our series were male and sustained their injury in a road traffic accident, consistent with the well-described predominance of high-energy trauma and male sex in published cohorts. Brautigan and Johnson, in their epidemiological review of knee dislocations, reported a male-to-female ratio of approximately 4:1 [6], and Vistisen et al., in a cross-sectional retrospective study, similarly described road traffic collisions as the predominant mechanism of injury in contemporary cohorts [4].

Anterior dislocation was the most frequent pattern in our series (57.1%), followed by lateral and posterior dislocation. This split largely tracks what has been reported elsewhere -- Kennedy’s original classification and the later multicentric work by Schenck both put anterior dislocation at the top of the list, accounting for roughly 30-50% of injuries and usually arising from a hyperextension mechanism [7,9]. Schenck also flagged posterior dislocation as the pattern carrying the greatest vascular risk, since axial loading drives the tibia straight back into the popliteal artery [9]; that association held in our series too, where the one confirmed vascular injury occurred in the patient with a posterior dislocation.

The vascular injury rate observed in this series (14.3%) is lower than the rate of up to 40% described by Wascher in his review of knee dislocation assessment and treatment [2], though it remains within the broad range reported across the literature and underscores the importance of a low threshold for vascular imaging even when overall numbers are small. Importantly, Twaddle et al., in a prospective MRI-correlated study, demonstrated that a palpable distal pulse does not reliably exclude a significant vascular injury, as collateral circulation around the knee can mask underlying arterial compromise [12]; Walker et al. similarly emphasized the limitations of pulse examination alone in their radiologic review of knee dislocation [11]. Serial examination and, where indicated, CT angiography therefore remain essential regardless of initial pulse status. In our series, the patient with absent pulses underwent prompt combined orthopaedic and vascular intervention and achieved limb salvage without amputation, consistent with Wascher’s later work on high-velocity knee dislocation, which showed that revascularization within a golden period of 6-8 hours substantially reduces amputation risk [14], a finding echoed by Teissier et al. in their series of knee dislocations with vascular injury [15].

External fixation was used in patients with higher-energy or unstable injury patterns, including the case with combined vascular injury. Ramírez-Bermejo et al., in a systematic review of external fixation following vascular injury in knee dislocation, described this approach as an effective means of providing provisional stability while permitting soft-tissue and vascular repair to proceed, deferring definitive ligament reconstruction to a staged setting [16]. Delayed ACL reconstruction, performed in two patients in our series following MRI-confirmed instability, aligns with the findings of Peskun et al. [17] and Plancher and Siliski [18], both of whom supported staged or delayed ligamentous reconstruction over prolonged immobilization, which they associated with inferior functional outcomes and stiffness. Almekinders and Dedmond similarly reported superior outcomes with operative treatment of the dislocated knee compared with nonoperative management alone [19].

The remaining patients in our series were managed conservatively with bracing and structured physiotherapy, reflecting an individualized approach based on post-reduction stability rather than a uniform operative protocol. This mirrors the approach described by Henrichs [20] and by Taylor et al. in one of the earliest large series of conservatively treated knee dislocations [21], both of whom supported conservative management in selected stable injuries while reserving operative ligament reconstruction for patients with demonstrable instability.

Functional and radiological follow-up in this series, while available for only a subset of patients, showed satisfactory restoration of knee mobility and ambulatory function without evidence of chronic instability, stiffness, or post-traumatic complications during the available follow-up period. This is consistent with the observations of Henrichs [20] and of Plancher and Siliski [18], both of whom reported that early, structured rehabilitation following either operative or conservative management improves functional recovery after knee dislocation.

Overall, our findings reinforce the central message of the existing literature. Robertson et al., in their review of knee dislocation [1], Wascher, in his widely cited treatment-principles review [2], and Boyce et al., in their guidance for the general orthopaedic surgeon [22], have each emphasized that outcome after knee dislocation is determined less by the direction of dislocation itself and more by the speed and rigor of the initial neurovascular assessment, the timeliness of vascular repair when indicated, and the appropriateness of the subsequent ligamentous management strategy.

Limitations 

Our study has some important limitations. With only seven patients treated at a single centre, the series is small, and we could not perform meaningful comparative or association analyses; the findings should therefore be read with caution before being applied more broadly. Follow-up also varied from patient to patient, as did the detail with which functional outcomes such as range of motion and gait were recorded. We did not have a standardized measure such as the Lysholm or International Knee Documentation Committee (IKDC) score for all cases, which makes it harder to compare our results with those of other studies. Peroneal nerve status was not consistently documented either, so nerve injuries may have been more common than our records suggest. And because the data were collected retrospectively, some clinical details had to be pieced together from imaging and operative notes rather than gathered prospectively under a set protocol.

## Conclusions

Traumatic knee dislocation is a high-risk injury that requires urgent reduction and systematic neurovascular assessment. In this seven-patient retrospective case series, one patient required urgent popliteal artery repair, and limb salvage was achieved; other patients were managed using external fixation, delayed ACL reconstruction, or conservative treatment according to clinical circumstances. The series underscores the importance of coordinated orthopaedic and vascular care while also demonstrating the limitations of drawing long-term functional or comparative treatment conclusions from a small, heterogeneous retrospective cohort.
